# Genetic analysis of Wnt/PCP genes in neural tube defects

**DOI:** 10.1186/s12920-018-0355-9

**Published:** 2018-04-04

**Authors:** Zhongzhong Chen, Yunping Lei, Xuanye Cao, Yufang Zheng, Fang Wang, Yihua Bao, Rui Peng, Richard H. Finnell, Ting Zhang, Hongyan Wang

**Affiliations:** 1 Obstetrics and Gynecology Hospital, State Key Laboratory of Genetic Engineering at School of Life Sciences, Institute of Reproduction and Development, Fudan University, Shanghai, 200011 China; 2 Key Laboratory of Reproduction Regulation of NPFPC, Collaborative Innovation Center of Genetics and Development, Fudan University, Shanghai, 200032 China; 3 Children’s Hospital and Institutes of Biomedical Sciences of Fudan University, Shanghai, China; 4 Departments of Molecular and Cellular Biology and Medicine, Baylor College of Medicine, Houston, TX 77030 USA; 5 Department of Pediatrics, Dell Pediatric Research Institute, University of Texas at Austin Dell Medical School, Austin, TX 78723 USA; 6 Beijing Municipal Key Laboratory of Child Development and Nutriomics, Capital Institute of Pediatrics, Beijing, 100020 China

**Keywords:** Neural tube defects, PCP (planar cell polarity), Wnt, Variant, CELSR

## Abstract

**Background:**

Mouse homozygous mutants in Wnt/planar cell polarity (PCP) pathway genes have been shown to cause neural tube defects (NTDs) through the disruption of normal morphogenetic processes critical to neural tube closure (NTC). Knockout mice that are heterozygotes of single PCP genes likely fail to produce NTD phenotypes, yet damaging variants detected in human NTDs are almost always heterozygous, suggesting that other deleterious interacting variants are likely to be present. Nonetheless, the Wnt/PCP pathway remains a genetic hotspot. Addressing these issues is essential for understanding the genetic etiology of human NTDs.

**Methods:**

We performed targeted next-generation sequencing (NGS) on 30 NTD-predisposing Wnt/PCP pathway genes in 184 Chinese NTD cases. We subsequently replicated our findings for the *CELSR1* gene in an independent cohort of 292 Caucasian NTD samples from the USA. Functional validations were confirmed using in vitro assays.

**Results:**

*CELSR1*, *CELSR2* and *CELSR3* genes were significantly clustered with rare driver coding mutations (q-value< 0.05) demonstrated by OncodriveCLUST. During the validation stage, the number of rare loss of function (LoF) variants in *CELSR1* was significantly enriched in NTDs compared with the LoF counts in the ExAC database (*p* < 0.001). Functional studies indicated compound heterozygote variants of *CELSR2* p.Thr2026Met and *DVL3* p.Asp403Asn result in down regulation of PCP signals.

**Conclusions:**

These data indicate rare damaging variants of the *CELSR* genes, identified in ~ 14% of NTD cases, are expected to be driver genes in the Wnt/PCP pathway. Compound damaging variants of *CELSR* genes and other Wnt/PCP genes, which were observed in 3.3% of the studied NTD cohort, are also expected to amplify these effects at the pathway level.

**Electronic supplementary material:**

The online version of this article (10.1186/s12920-018-0355-9) contains supplementary material, which is available to authorized users.

## Background

Neural tube defects (NTDs) resulting from the failure of neural tube closure (NTC) are among the most common and severe forms of developmental defects in humans. The complete closure of the neural tube requires the coordination of neural-plate apical constriction and convergent extension, both of which are directly regulated by the non-canonical Wnt/planar cell polarity (PCP) pathway [1]. Homozygous mouse mutants in the PCP pathway are known to cause NTDs through the disruption of NTC. Loss of function (LoF) alleles of the core components of the PCP pathway, including *Celsr1* [2] and *Vangl2* [3] produces craniorachischisis in mice. In addition, double but not single knockouts at *Fzd3* and *Fzd6* [4], as well as *Dvls* [5, 6] result in the NTD known as craniorachischisis in mice. In recent years, functional variants in PCP pathway genes have been identified in human NTDs, including: *CELSR1* [7, 8], *DVL2* [9], *VANGL1* [10], *VANGL2* [11], *SCRIB* [12], *LPR6* [13] and *FZD6* [14]. PCP genes appear to play an important role in the etiology of these human congenital malformations [15]. Besides these PCP components, alterations in Wnt signaling has previously been associated with NTDs [16] and Wnt signaling proteins also direct PCP signals in the vertebrate ectoderm [17]. The polarity of Prickle3/Vangl2 complex was disrupted by several Wnt antagonists in the ectoderm of early *Xenopus* embryos; while Wnt5a, Wnt11, and Wnt11b disrupted Prickle3/Vangl2 complexes in mid-gastrula embryos leading to the development of NTDs [17]. Despite recent advances in genomics and bioinformatics, the genetic contribution of Wnt/PCP pathway genes to human NTDs requires further interrogation. Unlike mutations in many PCP genes that cause NTDs in mice, the variants detected in human NTDs are overwhelmingly heterozygous. Although knock out heterozygotes of single inactivated mouse genes does not typically produce NTD phenotypes, disruption of multiple genes in an individual could produce devastating consequences on embryonic development, as observed in mice [18]. Rare damaging variants were used in identifying interactions with birth defects, such as craniosynostosis [19] and congenital heart disease [20]. These data suggest that the interactions between rare compound damaging variants may contribute to the etiology of NTDs in humans; however, there is a paucity of compound heterozygosity data on human NTDs.

This study analyzes a panel of Wnt/PCP genes in a population based cohort of human NTD samples. We found that all three *CELSR* genes (*CELSR1*, *CELSR2* and *CELSR3*) play driver roles in the etiology of human NTDs. In addition, this study is the first to identify compound PCP rare damaging variants combination in human NTDs, and these genetic combinations are expected to amplify effects at the pathway level and increase the risk of NTDs.

## Methods

### Human neural tube defects

Our study was conducted utilizing a two-stage design, and detailed diagnostic information on the patients is provided in an Additional file 1: Table S1. Subjects in the capture sequencing stage, consisting of 184 NTD subjects, were all ethnically Han Chinese, as previously described [11, 21, 22]. The Chinese NTD samples were collected from either aborted fetuses (23.4 ± 6.7 weeks) or children with spina bifida (6.4 ± 4.6 years). Subjects for the *CELSR1* validation study were primarily collected from Texas (292 cases, including 192 previously reported samples [7] and 100 additional samples) and the NTDs were diagnosed by trained neurosurgeons. The US NTD samples were collected from either new born or children with spina bifida with mean age 7.5 ± 5.4 years. Protocols were reviewed and approved by the Ethics Committee of the School of Life Sciences, Fudan University (EC#216). American NTD study protocols were reviewed and approved by the Institutional Review Board (IRB) of University of Texas at Austin (UT Austin IRB approval number: 2014120041). All of the samples were obtained with the appropriate parental informed consent.

### DNA sequencing, genotyping and data analysis

Capture baits were designed to cover all coding regions of 30 Wnt/PCP pathway genes based on KEGG databases [23], and the variants were detected using targeted capture and next generation DNA sequencing (NGS). Variant calling and annotation were conducted as presented in our previously published methods [8]. Based on results from NGS, rare variants were selected to characterize the patterns of Wnt/PCP pathway genes in human NTD cases using the following criteria: (i) minor allele frequency (MAF) in NTDs < 0.01; (ii) coding region and splice sites were included. All variants were further annotated with the Exome Aggregation Consortium (ExAC) [24]. The functional impact of missense mutations were predicted using Sorting Intolerant From Tolerant (SIFT) [25] and Polymorphism Phenotyping version 2 (Polyphen2) [26] via VEP [27].

### Plasmids

XE251 pCDNA3.1(zeo)-hDsh3-Flag (DVL3-Flag) was a gift from Randall Moon (Addgene plasmid # 16758) [28]. Plasmid pcDNA-3.0-CELSR2-GFP(CELSR2-GFP) (Clone ID: OHu24729) was purchased from Genescript. VANGL2-HA plasmid was house made by amplification of VANGL2 cDNA and cloning the VANGL2 cDNA into a pCMV-HA plasmid (CloneTech) as previously described [11]. DVL3 c.1207G > A(p.Asp403Asn) and CELSR2 c.6077C > T(p.Thr2026Met) were introduced into DVL3-Flag and CELSR2-GFP plasmids separately by GeneArt® Site-Directed Mutagenesis System (Life Technologies, Carlsbad, CA). M50 Super 8× TOPFlash was a gift from Randall Moon (Addgene plasmid # 12456) [29].

### Cell lines, culture conditions and transfection

HEK-293 T (human embryonic kidney), HeLa (human cervical cancer) were grown in DMEM (Gibco) supplemented with 10% heat-inactivated fetal bovine serum (FBS, Gibco). Cultures were maintained at 37 °C in a humidified atmosphere containing 5% CO_2_. Before transfection, cells were grown to 50-70% confluency. Cell transfection was carried out using Lipofectamine 2000 (ThermoFisher) according to the manufacturer’s protocol.

### Luciferase reporter assays

Topflash, a TCF/LEF reporter plasmid, was used for canonical Wnt pathway signaling detection [29]. AP1 reporter plasmid was used to measure activation by Jun-kinase, a downstream effector of the non-canoncial Wnt/PCP pathway signaling [13, 30]. For luciferase reporter assays in HEK-293 T cells, cells were transfected with pcDNA-3.0-Dvl3-Flag (Wildtype or Mutant), pcDNA-3.0-Celsr2-GFP (Wildtype or Mutant) and TOPFlash (Addgene plasmid ID:12456) [29] or pAP1-Luc (Agilent, Santa Clara, CA) [13] and Renilla-TK plasmid with indicated concentrations. Pools of cells were lysed with passive lysis buffer (Promega), and assayed for luciferase activity using the luciferase system (Promega). All luciferase reporter assays represent the mean ± standard error of the mean (SEM) from three independent measurements of cell pools. Three independent replicates were performed and all statistical tests were using two tailed t-test with *P* < 0.05 as the significance level.

### Co-immunoprecipitation assay

DVL3-Flag and VANGL2-HA were co-transfected into HEK-293 T cells and cell lysates were immunoprecipitated with Anti-Flag Dynabeads (ThermoFisher) overnight at 4 °C, and then washed three times with Lysis buffers and boiled with SDS loading buffer and subjected to Western blotting. Dvl3-Flag and Vangl2-HA were detected, as indicated.

### Sub-cellular localization detection

Cells were seeded onto 20 mm Glass Bottom Cell Culture Dish and transfected with Celsr2-GFP, Dvl3-Flag. Cells were washed in PBS prior to fixation in 4% formaldehyde. For Celsr2-GFP transfected cells, GFP was directly detected by a laser scanning confocal microscope (Leica,SP5). For Dvl3-Flag transfected cells, the cells were lysed and blocked by 1% BSA (Sigma Aldrich) for 1 h, after which time they were incubated overnight with 1% BSA and washed three times with cold PBS followed by an hour exposure to a FITC conjugated secondary antibody. Immunofluorescence was visualized by the EVOS cell imaging system (ThermoFisher Scientific).

### Statistical analysis

OncodriveCLUST [31], which identifies genes whose alterations tend to cluster in specific protein regions with respect to synonymous alterations [32], was used to identify driver gene mutations in the Wnt/PCP pathway in NTD cases. The Fisher’s exact test was employed for *CELSR1* LoF variants association analysis. Statistical analyses were performed using R software.

## Results

### Genes frequently affected by rare mutations in Wnt/PCP pathway using targeted next-generation sequencing

**Fig. 1:**
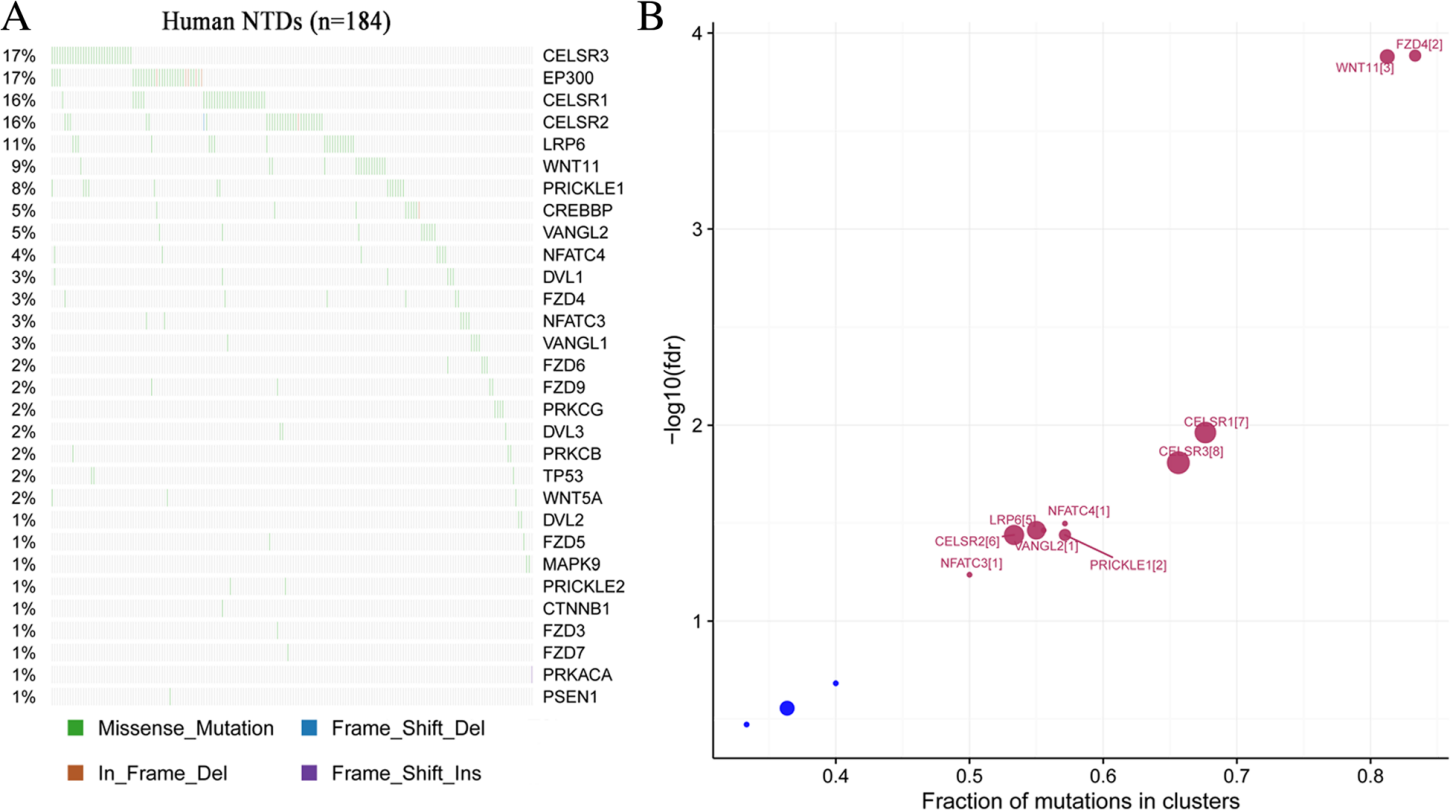
Frequently mutated genes and driver genes detected in human NTDs. **a** Frequently mutated genes across individual human NTDs. Wnt/PCP genes are shown according to decreasing mutational frequency. NTD samples are displayed as columns. Right bar depicts absolute number of rare mutations in each gene. **b** Driver genes identified in human NTDs using OncodriveCLUST

To better understand the genetic mechanisms underlying the etiology of human NTDs, we investigated the genetic contribution of 30 genes in the Wnt/PCP pathway. Among these 30 Wnt/PCP pathway genes, mouse mutants in 15 genes resulted in NTDs with variable phenotypes and penetrance [33]. There were 264 rare variants (MAF < 0.01) identified in the coding region and in splice sites of 30 Wnt/PCP pathway genes in the 184 human NTD samples. Among these rare variants, 255 were single nucleotide variants (SNVs) and 9 were small insertions or deletions (indels). 62 (33.7%) human NTDs carried 2 or more rare variants, whereas 122 (66.3%) human NTDs had only a single rare variant in the Wnt/PCP pathway. T > C and C > T were the two most common transitions identified in the Wnt/PCP pathway in human NTDs. The top four genes with the highest percentage of mutations in our human NTD samples are *CELSR3*, *EP300*, *CELSR1* and *CELSR2* (Fig. 1). This included 17% cases with *CELSR3*, 17% cases with *EP300*, 16% cases with *CELSR1* and 16% cases with *CELSR2* variants. With the exception of *CELSR1,* none of the other *CELSR* genes had previously been observed as NTD mouse candidate genes producing an abnormal phenotype when inactivated.

### Rare variants detected in *CELSR1-3* tend to be driver genes in Wnt/PCP pathway in human NTDs using OncodriveCLUST

In order to uncover the driver genes in the Wnt/PCP pathway responsible for NTDs, OncodriveCLUST [31] was used to examine those genes with significant positional clustering of mutations. It is a useful method to identify driver genes, whose biased mutations are clustered within the protein sequence for adaptive advances during evolution. Results showed that nine genes were significantly (q-value< 0.05) mutated in NTD cases, including: *FZD4*, *WNT11*, *CELSR1*, *CELSR3*, *NFATC4*, *LRP6*, *VANGL2*, *CELSR2* and *PRICKLE1* (Fig. 1b). Among the top four frequently mutated genes, all *CELSR* genes (*CELSR1*, *CELSR2* and *CELSR3*) were significantly (q-value< 0.05) mutated in NTDs, while *EP300* was not significantly mutated in NTD cases. Mutations in these 30 Wnt/PCP genes were frequently clustered at cadherin repeats, Wnt and strabismus domains (Additional file 2: Figure S1). Our previous study identified the missense mutation *CELSR1* p.Phe870Leu in the cadherin repeat domain that induced NTDs via disruption of both the PCP pathway and canonical WNT signaling [8]. This suggested that rare mutations in *CELSR* genes function as mutational hotspots in the etiology of NTDs. In order to validate our findings, we re-sequenced *CELSR1* in 292 NTDs (including 192 previously reported samples [7] and 100 additional samples) collected in the US. Three LoF variants (c.5050_5051insGT [7], c.5719_5720delTG [7] and c.4417A > T (p.Gln1473Ter)) were identified in the *CELSR1* gene. Compared with the LoF frequency from the ExAC database (13/60693), *CELSR1* LoF variants (3/292) in NTDs were significant enriched in our NTD cohort (Fisher test, *p* < 0.001).

### Detection of double damaging variants among *CELSR* genes and other Wnt/PCP genes in NTD cases

**Fig. 2:**
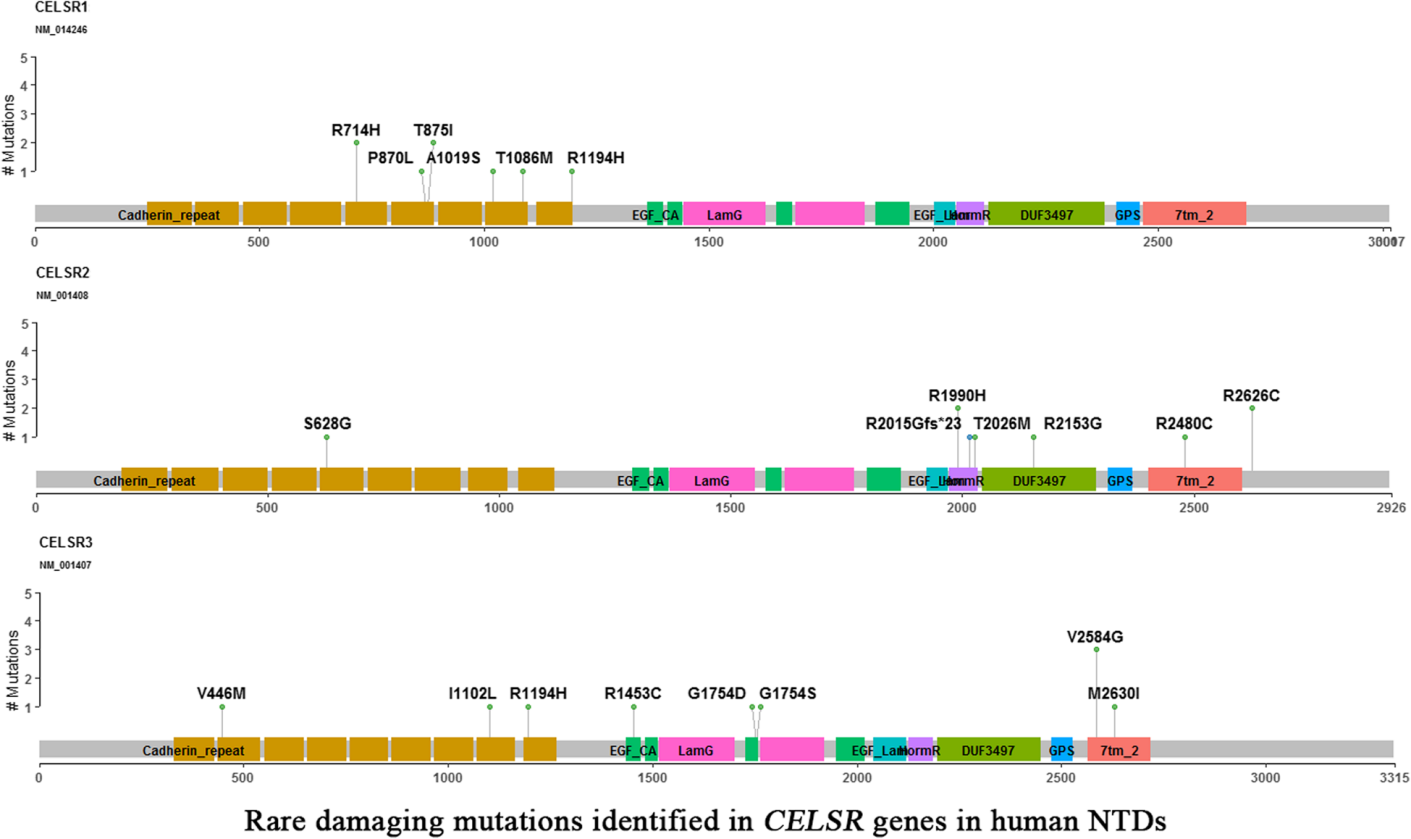
Mapping of rare damaging mutations in the *CELSR1*, *CELSR2*, *CELSR3* genes in human NTDs

There were 27 rare mutations of *CELSR* genes identified in 26 (14.1%) NTD cases that were predicted to be damaging variants by both PolyPhen-2 and SIFT (Fig. 2). These included eight (4.3%) cases with damaging variants of *CELSR1*, nine (4.9%) cases with damaging variants of *CELSR2,* and 10 (5.4%) cases with damaging variants of *CELSR3*. In addition, six (3.3%) cases were observed to have double Wnt/PCP heterozygous damaging mutants, and all of these cases have both damaging *CELSR* mutants concurrent with other Wnt/PCP mutations. These six cases were associated with diverse NTD phenotypes (Table 1). These double mutation cases included: case NTD_27 with *CELSR2* p.Thr2026Met and *DVL3* p.Asp403Asn, case NTD_39 with *CELSR2* p.Arg2480Cys and *FZD7* p.Leu383Gln, case NTD_56 with *CELSR2* p.Arg2626Cys and *FZD5* p.Trp242Leu, and case NTD_19 with *CELSR1* p.Thr1086Met and *VANGL1* p.Arg207His presented with spina bifida. Case NTD_122 with *CELSR2* p.Arg1990His and *CELSR3* p.Argy1194His had encephalocele. Case NTD_15 with *LRP6* p.Arg386Cys and *CELSR1* p.Arg714His had both anencephaly and spina bifida. Amongst the 12 damaging variants in these six cases, eight of them existed in the ExAC database with MAF < 0.001, while four variants were novel and did not exist in the ExAC database.

**Table 1:** Combined rare damaging variants among Wnt/PCP genes in human NTD samples

Sample	Gene	Variant	Chr.	Position^a^	Minor/major allele	Sex	Phenotype^b^	SIFT^c^	PP2^d^	MAF in ExAC^e^
NTD_27	*CELSR2*	p.Thr2026Met	1	109,810,233	T/C	M	SB	D	D	0.000009415
	*DVL3*	p.Asp403Asn	3	183,885,376	A/G			D	P	NA^f^
NTD_39	*CELSR2*	p.Arg2480Cys	1	109,813,177	T/C	M	SB	D	D	0.00003766
	*FZD7*	p.Leu383Gln	2	202,900,518	A/T			D	D	NA
NTD_122	*CELSR2*	p.Arg1990His	1	109,808,784	A/G	M	EC	D	P	0.0001318
	*CELSR3*	p.Arg1194His	3	48,696,487	T/C			D	D	0.00002825
NTD_15	*LRP6*	p.Arg386Cys	12	12,334,194	A/G	F	AE,SB	D	D	0.000273
	*CELSR1*	p.Arg714His	22	46,930,927	T/C			D	D	NA
NTD_56	*CELSR2*	p.Arg2626Cys	1	109,814,294	T/C	NA	SB	D	P	0.0004425
	*FZD5*	p.Trp242Leu	2	208,632,739	A/C			D	D	NA
NTD_19	*VANGL1*	p.Arg207His	1	116,206,697	A/G	F	SB	D	D	0.00001883
	*CELSR1*	p.Thr1086Met	22	46,929,811	A/G			D	D	0.0001318

^a^Positions are given in bp from GRCh37

^b^SB, spinabifida; EC, encephalocele; AE, anencephaly

^c^SIFT predictions: D, deleterious

^d^PolyPhen2 (PP2) predictions: D, probably damaging; P, possibly damaging

^e^MAF from Exome Aggregation Consortium (ExAC) database

^f^Not available

### Functional validation of *CELSR2* p.Thr2026Met and *DVL3* p.Asp403Asn

**Fig. 3:**
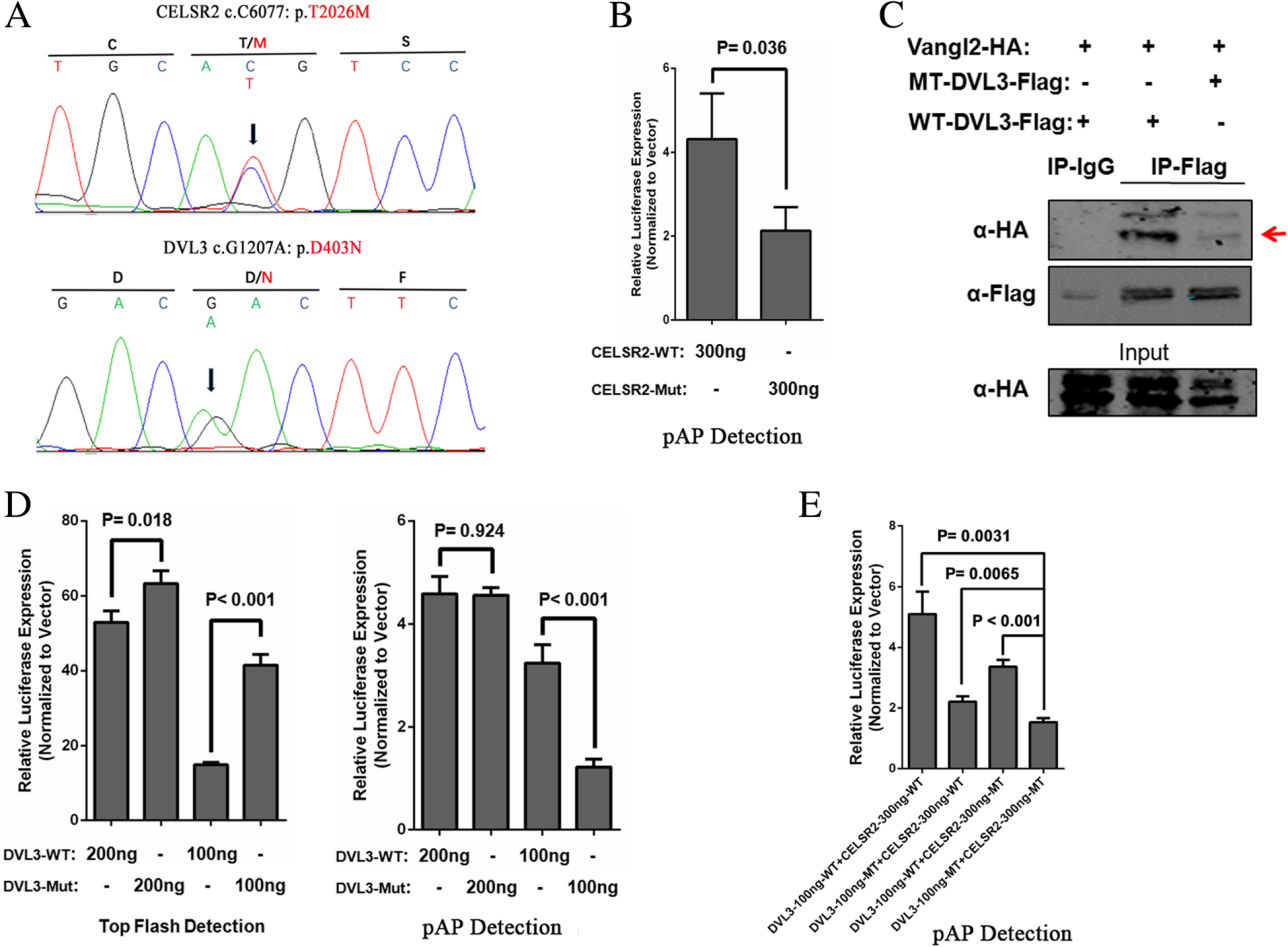
Combination of rare damaging mutations in *CELSR2* p.Thr2026Met and *DVL3* p.Asp403Asn in an individual showed the combined effect on down regulating PCP pathway. **a** Confirmation of *CELSR2* p.Thr2026Met and *DVL3* p.Asp403Asn by sanger sequencing. **b** *CELSR2* p.Thr2026Met transfection downregulated PCP signal in HEK293T cell line. **c** *DVL3* p.Asp403Asn disrupted *DVL3* interaction with *VANGL2*. **d** *DVL3* p.Asp403Asn transfection affects canonical Wnt and PCP signaling in HEK293T cell line. **e** *DVL3* p.Asp403Asn and *CELSR2* p.Thr2026Met both down regulated PCP signal, the combination of *DVL3* p.Asp403Asn and *CELSR2* p.Thr2026Met had lowest PCP signal

To determine the combined effect of compound heterozygosity damaging variants on PCP signaling, the functional impact of *CELSR2* p.Thr2026Met and *DVL3* p.Asp403Asn on canonical Wnt signaling and PCP signaling was examined in case NTD_27. These two heterozygous variants were validated by Sanger sequencing (Fig. 3a). Neither of these two variants affected the protein subcellular localization (Additional file 3: Figure S2). *CLESR2* p.Thr2026Met downregulated the PCP pathway signaling in our pAP1-Luc luciferase assay (Fig. 3b). *DVL3* p.Asp403Asn reduced interactions between DVL3 and VANGL2 (Fig. 3c). The *DVL3* p.Asp403Asn affected both canonical and non-canonical Wnt signaling in our luciferase assay (Fig. 3d). It upregulated canonical Wnt signaling in our TopFlash assay and down regulated non-canonical Wnt/PCP signaling in our pAP1-Luc luciferase assay when 100 ng plasmid was transfected. The two variants demonstrated a combined effect on down regulating PCP pathway signals in our pAP1-Luc luciferase assay (Fig. 3e).

## Discussion

Mutations in over 300 mouse genes are associated with diverse NTD phenotypes [33], and a large number of these genes cluster in the PCP pathway [18, 34]. Recent mouse genetic studies have identified that double-heterozygote combinations of *Vangl2*^*LP*^, *Scrib*^*Crc*^ and *Celsr1*^*Crsh*^ are associated with a variety of NTDs, including anencephaly and spina bifida [18]. While homozygous PCP knockout mice demonstrated an NTD phenotype, single PCP heterozygote mutant mice likely fail to produce NTD phenotypes. Instead, double or multiple PCP genes in heterozygous combinations appears to be required in order to express the NTD phenotype. We propose that compound heterozygosity of genes in the PCP pathway in human populations, where multiple PCP gene functional variants work together in the heterozygous state, can result in NTDs. Previously reported human NTDs with missense PCP mutations were all single heterozygotes, suggesting that the ‘second’ or ‘the other’ deleterious interacting variant might be present [15]. Understanding the genetic mechanisms of Wnt/PCP pathway is essential for appreciating the genetic architecture of human NTDs. In this study, we focused on the genetic hotspots of rare coding mutants in the Wnt/PCP pathway genes, in regard to determining their genetic impact on susceptibility to NTDs. Overall, rare coding mutations were observed to be frequently mutated in *CELSR1-3* genes. Moreover, we identified 84 (46%) cases with rare missense mutations, and 26 (14.1%) cases with rare damaging missense mutations in *CELSR* genes, respectively. Importantly, we determined that six (3.3%) of these cases had both damaging *CELSR* mutations and additional deleterious mutations of other Wnt/PCP genes, which were associated with diverse NTD phenotypes in humans.

These findings suggest that rare mutations of *CELSR* genes are identified as driver genes in human NTDs. *CELSR*, or *Flamingo* (*Fmi*) genes are a special subgroup of adhesion G protein-coupled receptors (GPCRs) that have three homologs, *CELSR1*, *CELSR2* and *CELSR3*. *CELSR* genes were first identified in *Drosophila* for regulating planar cell polarity in the wing [35]. Robinson and co-workers detected missense variants in *CELSR1* increased risk of craniorachischisis in humans [36]. Allache and co-workers identified pathogenic *CELSR1* mutations in 2.9% of Italian and Canadian NTD cohort [37]. Lei and colleagues detected deleterious *CELSR1* variants in about 3% of spina bifida cases collected in the U.S. cohort [7]. In contrast to *CELSR1*, the genetic evidence of *CELSR2* and *CELSR3* contributing to NTD risk was previously lacking. Our previous study identified the missense mutation *CELSR1* p.Phe870Leu in the cadherin repeat domain induced NTDs via disruption of both the PCP pathway and canonical WNT signaling [8]. It is implied that *CELSR2* and *CELSR3*, clustered at cadherin repeat domain, may also contribute the human NTD risk. In the present study, we found that *CELSR2* p.Thr2026Met, which is located in the HormR domain, affected PCP signaling in the HEK293T cell line (Fig. 3). Taken together, we found that in addition to *CELSR1*, both *CELSR2* and *CELSR3* also contribute to genetic etiology of human NTDs.

Previous studies reported that the damaging mutations of *CELSR1* are associated with diverse NTD phenotypes in humans, such as craniorachischisis [36] and spina bifida [7]. Given the different phenotypes, these data suggests that human NTDs could be caused by compound mutant heterozygosity. *Celsr2* or *Celsr3* regulate forebrain wiring in a Vangl-independent manner and inactivation of *Celsr2* or *Celsr3* produces abnormal brain morphology but does not produce NTDs in mice [38], also implicating that other unidentified molecules may participate in the genetic interactions leading to human NTDs. The present study further supported this notion by examining the damaging mutations of *CELSR* genes and other Wnt/PCP genes. Six (3.3%) of NTD cases were identified to have double damaging variants that included *CELSR* genes and other Wnt/PCP genes, such as *CELSR2* p.Thr2026Met and *DVL3* p.Asp403Asn. This combination was determined to have the lowest PCP signal in vitro (Fig. 3). In addition, among these eight genes involved in the double damaging heterozygous mutations combinations (Table 1), damaging variants of the *CELSR1* [7, 8], *VANGL1* [10], *LPR6* [13] and *Dvl3* [5] are known to be associated with NTDs, indicating that compound heterozygote damaging variants, *LRP6* p.Arg386Cys and *CELSR1* p.Arg714His (case NTD_15), *CELSR1* p.Thr1086Met and *VANGL1* p.Arg207His (case NTD_19) may increase human NTD risk. As other combinations of double damaging mutations might also expand the genetic risk factors potentially associated with NTDs, further experiments are warranted to assess their contribution to the risk for human NTDs. Altogether, we added to our understanding of the role of *CELSR2* and *CELSR3* in human NTDs, and established that the combination of rare damaging mutations in *CELSR* genes and other Wnt/PCP variants in an individual could increase human NTD risk. Different combinations are associated with diverse NTD phenotypes, including spina bifida, encephaloceles, and the combined phenotype of anencephaly and spina bifida (Table 1). Our findings suggested that combinations of multiple deleterious heterozygous mutations in individuals might better explain the etiology of NTDs in humans. There are two major limitations in this study. First, we found *CELSR* genes as the driver genes in NTDs using OncoDriveClust [31] that is often used for identifying cancer-driving genes. Although all the compound heterozygote mutants found contain the *CELSR* genes, suggesting that *CELSR* genes are possibly the genetic hotspots in NTDs, the validity of this method needs further evaluation in human NTD cases. Second, further studies of whole genome or exome sequencing with larger sample sizes are warranted to better appreciate the genetic contributions of *CELSR* genes and additional PCP gene-gene interaction in the development of NTDs.

## Conclusions

In conclusion, this study demonstrates that damaging mutations of *CELSR* genes are genetic hotspots in the development of NTDs in our human population. We also emphasized that the increased risk of double damaging variants of *CELSR* genes and other Wnt/PCP genes, which was observed in 3.3% of our human NTD samples. The variable outcomes of mutations in *CELSR* genes that interact with other Wnt/PCP genes are direct evidence linking these genes to the etiology of human NTDs, indicating that multiple genetic mutations are important for understanding the genetic etiology of human NTDs.

## Additional files

Additional file 1: **Table S1.** Demographic characteristics in NTD cohorts. (DOCX 17 kb)

Additional file 2: **Figure S1.** Most frequent Pfam domains affected by Wnt/PCP genes in human NTD samples. (TIFF 2976 kb)

Additional file 3: **Figure S2.** *CELSR2* p.Thr2026Met and *DVL3* p.Asp403Asn did not affect the protein subcellular localization. (A) *CELSR2* p.Thr2026Met did not affect *CELSR2* subcellular localization in HEK293T & MDCKII cells transfected with CELSR2-GFP and CELSR2 (p.Thr2026Met)-GFP expression plasmids. (B) *DVL3* p.Asp403Asn did not affect *DVL3* subcellular localization in HEK293T. (TIFF 4540 kb)
