# Correction to: Genetic analysis of Wnt/PCP genes in neural tube defects

**DOI:** 10.1186/s12920-021-00881-7

**Published:** 2021-01-27

**Authors:** Zhongzhong Chen, Yunping Lei, Xuanye Cao, Yufang Zheng, Fang Wang, Yihua Bao, Rui Peng, Richard H. Finnell, Ting Zhang, Hongyan Wang

**Affiliations:** 1 Obstetrics and Gynecology Hospital, State Key Laboratory of Genetic, Engineering at School of Life Sciences, Institute of Reproduction and Development, Fudan University, Shanghai, 200011 China; 2 Key Laboratory of Reproduction Regulation of NPFPC, Collaborative Innovation Center of Genetics and Development, Fudan University, Shanghai, 200032 China; 3 Children’s Hospital and Institutes of Biomedical Sciences of Fudan University, Shanghai, China; 4 Departments of Molecular and Cellular Biology and Medicine, Baylor College of Medicine, Houston, TX 77030 USA; 5 Department of Pediatrics, Dell Pediatric Research Institute, University of Texas at Austin Dell Medical School, Austin, TX 78723 USA; 6 Beijing Municipal Key Laboratory of Child Development and Nutriomics, Capital Institute of Pediatrics, Beijing, 100020 China

## Correction to: BMC Med Genomics (2018) 11, 38 10.1186/s12920-018-0355-9

Following publication of the original article [1], it was reported that the article published without the proper disclosure.

Dr. Finnell formerly held a leadership position with the now dissolved TeratOmic Consulting LLC. He also receives travel funds to attend editorial board meetings of the *Journal of Reproductive and Developmental Medicine* published out of the Red Hospital of Fudan University.
