# Supplementary material for: Genetic analysis of Wnt/PCP genes in neural tube defects
Source: BMC Med Genomics. 2018 Apr 4;11:38. doi: 10.1186/s12920-018-0355-9 (PMC5885375; doi:10.1186/s12920-018-0355-9)
Supplement: Supplementary file 1 — Table S1. Demographic characteristics in NTD cohorts. (DOCX 17 kb) [file 12920_2018_355_MOESM1_ESM.docx]

**Table S1.** Demographic characteristics in NTD cohorts.

| Variable | China Cohort | US Cohort |
| --- | --- | --- |
| Case # | 184 | 292 |
| Age: weeks/years (mean±SD) | |  |
|  | 23.4±6.7wks | New Born |
|  | 6.4±4.6yrs | 7.5±5.4yrs |
| Gender: |  |  |
| Male | 68 (37%) | 94 (32%) |
| Female | 92 (50%) | 172 (59%) |
| Unknown | 24 (13%) | 26 (9%) |
| AE | 36 (20%) |  |
| CRS | 12 (7%) |  |
| EC | 32 (17%) |  |
| EX | 2 (1%) |  |
| SB | 91 (49%) | 292 (100%) |
| Unknown | 11 (6%) |  |

**AE**, anencephaly; **CRS**, craniorachischisis; **EC**, encephalocele;

**EX**, Exencephaly; **SB**, spina bifida
