# Supplementary figures and images for: Genetic analysis of Wnt/PCP genes in neural tube defects
Source: BMC Med Genomics. 2018 Apr 4;11:38. doi: 10.1186/s12920-018-0355-9 (PMC5885375; doi:10.1186/s12920-018-0355-9)

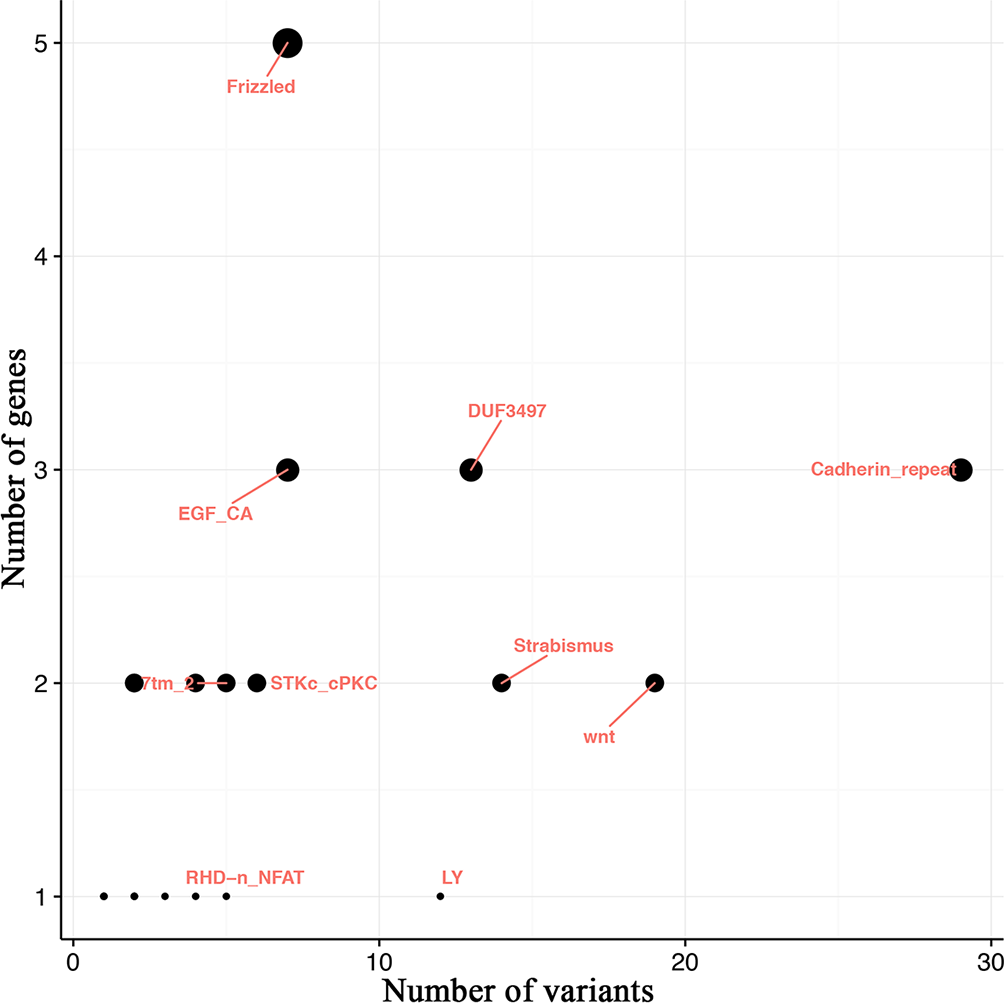

Supplement: Supplementary file 2 — Figure S1. Most frequent Pfam domains affected by Wnt/PCP genes in human NTD samples. (TIFF 2976 kb) [file 12920_2018_355_MOESM2_ESM.tif]

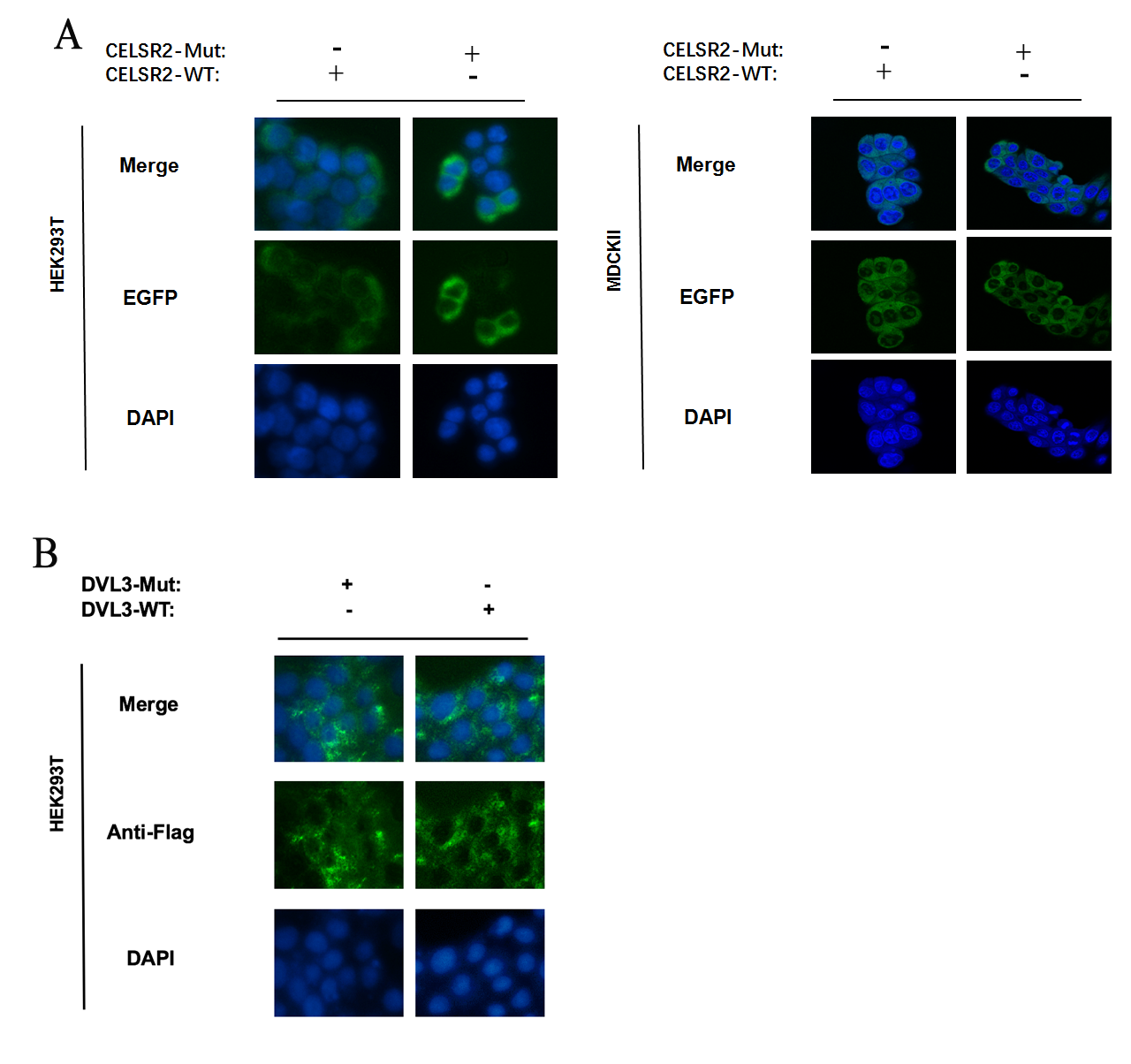

Supplement: Supplementary file 3 — Figure S2. CELSR2 p.Thr2026Met and DVL3 p.Asp403Asn did not affect the protein subcellular localization. (A) CELSR2 p.Thr2026Met did not affect CELSR2 subcellular localization in HEK293T & MDCKII cells transfected with CELSR2-GFP and CELSR2 (p.Thr2026Met)-GFP expression plasmids. (B) DVL3 p.Asp403Asn did not affect DVL3 subcellular localization in HEK293T. (TIFF 4540 kb) [file 12920_2018_355_MOESM3_ESM.tif]
